# Citrus CitNAC62 cooperates with CitWRKY1 to participate in citric acid degradation via up-regulation of *CitAco3*

**DOI:** 10.1093/jxb/erx187

**Published:** 2017-06-15

**Authors:** Shao-jia Li, Xue-ren Yin, Wen-li Wang, Xiao-fen Liu, Bo Zhang, Kun-song Chen

**Affiliations:** 1 College of Agriculture and Biotechnology, Zhejiang University, Zijingang Campus, Hangzhou, China; 2 Zhejiang Provincial Key Laboratory of Horticultural Plant Integrative Biology, Zhejiang University, Zijingang Campus, Hangzhou, China; 3 The State Agriculture Ministry Laboratory of Horticultural Plant Growth, Development and Quality Improvement, Zhejiang University, Zijingang Campus, Hangzhou, China

**Keywords:** Aconitase, CitNAC62, citric acid, CitWRKY1, protein–protein interaction, transcriptional regulation

## Abstract

Citric acid is the predominant organic acid of citrus fruit. Degradation of citric acid occurs during fruit development, influencing fruit acidity. Associations of *CitAco3* transcripts and citric acid degradation have been reported for citrus fruit. Here, transient overexpression of *CitAco3* significantly reduced the citric acid content of citrus leaves and fruits. Using dual luciferase assays, it was shown that CitNAC62 and CitWRKY1 could transactivate the promoter of *CitAco3*. Subcellular localization results showed that CitWRKY1 was located in the nucleus and CitNAC62 was not. Yeast two-hybrid analysis and bimolecular fluorescence complementation (BiFC) assays indicated that the two differently located transcription factors could interact with each other. Furthermore, BiFC showed that the protein–protein interaction occurred only in the nucleus, indicating the potential mobility of CitNAC62 in plant cells. A synergistic effect on citrate content was observed between CitNAC62 and CitWRKY1. Transient overexpression of *CitNAC62* or *CitWRKY1* led to significantly lower citrate content in citrus fruit. The combined expression of *CitNAC62* and *CitWRKY1* resulted in lower citrate content compared with the expression of *CitNAC62* or *CitWRKY1* alone. The transcript abundance of *CitAco3* was consistent with the citrate content. Thus, we propose that a complex of CitWRKY1 and CitNAC62 contributes to citric acid degradation in citrus fruit, potentially via modulation of *CitAco3*.

## Teaser

Two novel transcription factors, CitNAC62 and CitWRKY1, were involved in citric acid degradation in citrus fruit, potentially via enhanced expression of *CitAco3*.

## Introduction

Organic acids, including quinic, citric, malic, and oxalic acids, are present in most plants and vary among species, organ, and tissue types, developmental stages, and environmental conditions (Badia *et al.*, 2015). In Arabidopsis, organic acids influence carbohydrate perception in germinating seedlings (Hooks *et al.*, 2004), fumarate accumulation plays an essential role in low temperature sensing (Dyson *et al.*, 2016), malate is involved in cellular pH regulation and stomatal movement (Hurth *et al.*, 2005; Lee *et al.*, 2008), and citrate contributes to metal resistance in plant roots (Wang *et al.*, 2016).

Organic acid metabolism and degradation have been widely studied. For instance, *MxCS2*, a gene encoding a putative citrate synthase in *Malus xiaojinensis*, was introduced into Arabidopsis, resulting in increased citrate content (Han *et al.*, 2015). In contrast, inhibition of aconitase activity resulted in the accumulation of citrate (Gupta *et al.*, 2012; Hooks *et al.*, 2014). In addition to biosynthesis and degradation, some transporters, including a tonoplast dicarboxylate transporter (AttDT) (Hurth *et al.*, 2005), aluminum-activated malate transporter (ALMT) (Kovermann *et al.*, 2007), and some V-ATPase/V-PPase genes (Li *et al.*, 2016; Hu *et al.*, 2016), also influence organic acid content in plants. In citrus, a vacuolar citrate/H^+^ symporter was isolated that could mediate citrate efflux and play a role in citric acid homeostasis (Shimada *et al.*, 2006). In recent years, some transcription factors have been demonstrated to have important roles in the regulation of organic acids. In Arabidopsis, WRKY46 functions as a transcriptional repressor of *ALMT1*, regulating aluminum-induced malate secretion (Ding *et al.*, 2013). In tomato fruits, overexpression of *SlAREB1* resulted in increased citric and malic acid contents, and the expression of the mitochondrial citrate synthase gene (*mCS*) was up-regulated (Bastías *et al.*, 2011), while *CgDREB*-overexpressing tomato fruits showed higher levels of organic acids (Nishawy *et al.*, 2015). However, transcriptional regulatory information is still very limited.

In citrus fruit, especially acidic varieties, citric acid is the predominant organic acid, accounting for more than 90% of total organic acids (Albertini *et al.*, 2006; Baldwin *et al.*, 2014). The difference in the acidity of various citrus fruits at the commercial mature stage is due to expansion of the fruit, citrate synthesis and vacuole storage, and is also largely determined by the degradation pathway, including the gamma-aminobutyric acid (GABA) shunt and the glutamine and acetyl-CoA pathways (Katz *et al.*, 2011; Walker *et al.*, 2011; Lin *et al.*, 2015). Among these, the GABA shunt was considered to be the dominant pathway; the first step of this pathway is the conversion of citrate to isocitrate by aconitase (Terol *et al.*, 2010). In citrus fruits, inhibition of mitochondrial aconitase activity contributes to acid accumulation, and increasing cytosolic aconitase activity reduces the citrate level toward fruit maturation (Degu *et al.*, 2011; Sadka *et al.*, 2000). Transcript analysis from multiple sources indicated that *CitAco3* is negatively correlated with citric acid content in citrus fruit and *CitAco3* may contribute to citrate degradation (Chen *et al.*, 2012, 2013). However, understanding of the molecular basis of fruit citrate degradation has been limited to transcript analysis, including *CitAco3* and the other structural genes. Because of the difficulty of producing transgenic citrus material, the *in planta* roles of these genes in citrate degradation, and the *in vivo* mechanisms regulating their transcripts, remain unknown.

In the present research, gene expression and partial functional verification of *CitAco3* in relation to citrate degradation were studied. In order to understand the regulation of *CitAco3* expression, a set of 16 transcription factors was isolated on the basis of their co-expression with *CitAco3*. The potential regulatory roles of the transcription factors were investigated and two of them showed transactivation activity of the *CitAco3* promoter. In addition, the interaction and synergistic effects of two transcription factors, protein–protein interaction, and the possible movement of transcription factors within the plant cell were evaluated with regard to citrate degradation.

## Materials and methods

### Plant materials

Ponkan (*Citrus reticulata* Blanco cv. Ponkan) fruits received from a commercial orchard in Quzhou, Zhejiang, China, were used in this study. Fruits of uniform size and appearance were collected at each sampling point, from six different trees. Sampling points were at 60, 90, 120, 150, and 180 days after full blossom (DAFB). The flesh was frozen in liquid nitrogen and stored at −80 °C for further experiments.

### Citric acid measurement

The citric acid content of Ponkan fruits and leaves was measured according to Lin *et al.* (2015). Fruits (0.1 g) and leaves (0.05 g) were ground in liquid nitrogen and extracted with 1.4 ml methanol at 70 °C for 15 min, and then centrifuged at 10000 *g*. The upper phase was removed and stored at −80 °C until analysis. Aliquots of 100 μl upper phase were dried in a vacuum. The residue was dissolved in 40 μl 20 mg ml^−1^ pyridine methoxyamine hydrochloride, and incubated at 37 °C for 1.5 h. The sample was then treated with 60 μl Bis(trimethylsilyl)trifluoroacetamide (1% trimethylchlorosilane) at 37 °C for 30 min. Ribitol (20 μl, 0.2 mg ml^−1^) was added to each sample as an internal standard. A 1 μl aliquot of each sample was absorbed with a split ratio of 1:1 and injected into a GC-MS fitted with a fused-silica capillary column (30 m×0.25 mm internal diameter, 0.25 μm DB-5 MS stationary phase). The injector temperature was 250 °C and the helium carrier gas had a flow rate of 1.0 ml min^−1^. The column temperature was held at 100 °C for 1 min, increased to 184 °C at a rate of 3 °C min^−1^, then increased to 230 °C at a rate of 15 °C min^−1^ and held for 1 min. The MS operating parameters were as follows: ionization voltage 70 eV, ion source temperature as 230 °C, and interface temperature 280 °C.

### RNA extraction and cDNA synthesis

Total RNA was extracted from frozen tissues according to the protocol described by Chen *et al.* (2012). The genomic DNA in total RNA was degraded with RNase-free DNase I (Ambion). First-strand cDNA synthesis was initiated with 1.0 μg DNA-free RNA and GoScript™ Reverse Transcriptase (Promega) following the manufacturer’s protocol. Ten-fold diluted cDNA was used as the template for quantitative real-time PCR analysis. RNA extraction and cDNA synthesis were performed with three biological replicates for each sampling point.

### Real-time PCR

The PCR mixture (20 μl total volume) comprised 10 μl Lightcycler480 SYBR Green I Master (Roche), 1 μl of each primer (10 mM), 2 μl diluted cDNA and 6 μl PCR-grade H_2_O. PCR was performed on a Lightcycler 480 instrument (Roche), initiated by 5 min at 95 °C, followed by 50 cycles of 95 °C for 10 s, 60 °C for 10 s, and 72 °C for 15 s, and completed with a melting curve analysis program. No-template controls and melting curve analyses were included in every reaction. Citrus *actin* (XM_006464503) was used as a control to quantify cDNA abundance (Chen *et al.*, 2012). The sequences of the primers used are described in Supplementary Table S1 at *JXB* online.

### Dual luciferase assays

Dual luciferase assays were performed as described in our previous reports (Xu *et al.*, 2014). The promoter of the *CitAco3* gene was amplified with the primers described in Supplementary Table S2. Full-length transcripts of the transcription factors (Table 1) were inserted into the pGreen II 0029 62-SK vector (SK) with the primers described in Supplementary Table S2, while the promoter was inserted into the pGreen II 0800-LUC vector. Details of the vectors are given in Hellens *et al* (2005).

**Table 1.** Transcription factors highly correlated with CitAco3 from RNA-Seq data

Gene ID	Gene	*P*	*r*
Ciclev10021941m	Basic helix-loop-helix (bHLH) DNA-binding superfamily protein 1	0.987446	1.6 × 10^–5^
Ciclev10019368m	NAC domain-containing protein 62	0.982838	4.03 × 10^–5^
Ciclev10001803m	Myb domain protein 52	0.971244	0.000182
Ciclev10006902m	Heat shock transcription factor A6B	0.967535	0.000259
Ciclev10031361m	bZIP transcription factor family protein 1	0.946361	0.001093
Ciclev10009050m	MYB-like 102	0.944633	0.001196
Ciclev10024061m	WRKY family transcription factor 1	0.918417	0.003529
Ciclev10021312m	NAC domain-containing protein 74	0.913783	0.004108
Ciclev10000612m	NAC domain-containing protein 17	0.897303	0.006609
Ciclev10005649m	Heat shock transcription factor B3	0.896847	0.006688
Ciclev10015986m	Myb domain protein 62	0.89587	0.00686
Ciclev10028428m	bZIP transcription factor family protein 3	0.888003	0.008341
Ciclev10009361m	Ethylene response factor 7	0.883772	0.009208
Ciclev10005233m	Basic helix-loop-helix (bHLH) DNA-binding superfamily protein 2	0.869383	0.012537
Ciclev10020717m	NAC domain-containing protein 47	0.864434	0.013821
Ciclev10025940m	TGA1 bZIP transcription factor family protein	0.824062	0.027005

*P*, significance level; *r*, correlation coefficient.

All the constructs were electroporated into *Agrobacterium tumefaciens* GV3101. The dual luciferase assays were performed with tobacco (*Nicotiana benthamiana*) leaves. *Agrobacterium* cultures were prepared with infiltration buffer (10 mM MES, 10 mM MgCl_2_, 150 mM acetosyringone, pH 5.6) to an OD_600_ of 0.7–1.0. Mixtures of cultures of *Agrobacterium* expressing transcription factors (1 ml) and the promoter (100 µl) were infiltrated into tobacco leaves using needleless syringes. The tobacco plants were grown in a glasshouse with daylight extension to 16 h. Three days after infiltration, firefly luciferase and renilla luciferase were assayed using dual luciferase assay reagents (Promega). For each transcription factor–promoter interaction, three independent experiments were performed (five biological replicates in each experiment).

### Subcellular localization analysis

35S-*CitNAC62*-GFP and 35S-*CitWRKY1*-GFP were transiently expressed in tobacco leaves by *Agrobacterium*-mediated infiltration (GV3101) according to previous reports with some modification (Xu *et al.*, 2014). The green fluorescent protein (GFP) fluorescence of tobacco leaves was imaged 3 d after infiltration using a Zeiss LSM710NLO confocal laser scanning microscope. Primers used for GFP construction are described in Supplementary Table S3.

### Yeast two-hybrid assay

Protein–protein interactions were investigated in yeast with the DUAL hunter system (Dual-systems Biotech, Switzerland). Full-length coding sequences of *CitWRKY1* were cloned into the pDHB1 vector as bait, and the full length of *CitNAC62* was cloned into pPR3N vector as prey. The primers used for vector construction are described in Supplementary Table S4.

All constructs were transformed into the yeast strain NMY51 according to the manufacturer’s instructions. The assays were performed with different media: (i) SD medium lacking Trp and Leu (DDO); (ii) SD medium lacking Trp, Leu, His, and Ade (QDO); and (iii) SD medium lacking Trp, Leu, His, and Ade, and supplemented with 60 mM 3-amino-1,2,4-triazole (QDO+3AT). Auto-activations were tested with empty pPR3-N vectors and target genes with pDHB1, which were co-transformed in NMY51 and plated on QDO. Auto-activations were indicated by the presence of colonies. Protein–protein interaction assays were performed with co-transformation of *CitNAC62* in pPR3N and *CitWRKY1* in pDHB1. The presence of colonies in QDO and QDO+3AT indicated a protein–protein interaction.

### Bimolecular fluorescence complementation assay

Full-length *CitNAC62* and full-length *CitWRKY1* were cloned into either C-terminal or N-terminal fragments of yellow fluorescent protein (YFP) vectors (Sainsbury *et al.*, 2009). Primers used are listed in Supplementary Table S4. All constructs were transiently expressed in tobacco leaves by *Agrobacterium*-mediated infiltration (GV3101) based on previous reports with some modifications (Li *et al.*, 2016). The YFP fluorescence of tobacco leaves was imaged 3 d after infiltration using a Zeiss LSM710NLO confocal laser scanning microscope.

### Transient overexpression in citrus leaves and fruits

Full-length coding sequences of target genes (*CitAco3*, *CitNAC62*, and *CitWRKY1*) were amplified with primers (listed in Supplementary Table S5) and inserted into the SK vector. Information regarding the SK vector is given in Hellens *et al.* (2005). The constructs were electroporated into *Agrobacterium* GV3101. For transient overexpression in leaves, *Agrobacterium* cultures carrying empty vector (SK) or target genes were infiltrated into different sides of the same leaf. In fruit, two uniform sections were chosen from one Ponkan fruit, and were infiltrated with *Agrobacterium* cultures carrying empty vector (SK) or target genes, respectively. Five days after infiltration, the infiltrated leaves and sections were sampled and used for citric acid analysis.

### Statistical analysis

Least significant difference (LSD) was calculated by using DPS 7.05 (Zhejiang University, Hangzhou, China). The statistical significance of differences was calculated using Student’s *t*-test. Figures were drawn using Origin 8.0 (Microcal Software Inc.).

## Results

### *Association between* CitAco3 *and citrate degradation*

**Fig. 1.**
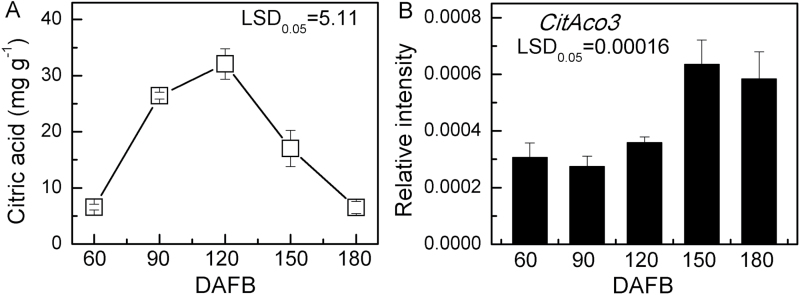
Changes in (A) the citric acid content and (B) the expression of *CitAco3* in the flesh of Ponkan fruits during fruit development. DAFB, days after full blossom. Error bars represent SE (*n*=3).

The correlation of *CitAco3* expression and citric acid degradation has been widely supported (Chen *et al.*, 2012; Lin *et al.*, 2015). In the present study, we found that *CitAco3* is more abundant in late developmental stages (150 and 180 DAFB), when the fruit citric acid decreased from a peak of 32.07 mg g^−1^ at 120 DAFB to 6.51 mg g^−1^ at 180 DAFB (Fig. 1A, B).

**Fig. 2.**
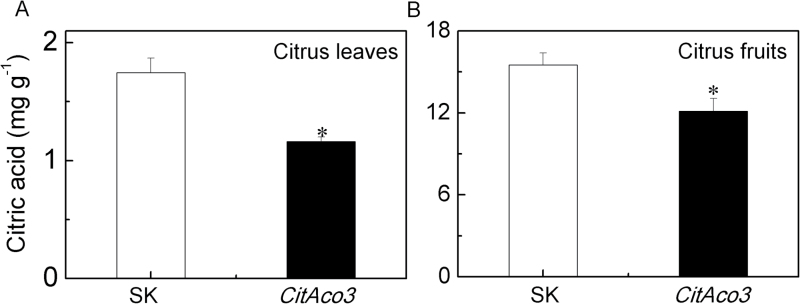
Transient overexpression of *CitAco3* in (A) citrus leaves and (B) fruits. The *CitAco3* gene was driven by the CaMV 35S promoter. SK represents empty vector. Citric acid was analyzed at 5 d after infiltration. Error bars indicate SE from five biological replicates. *Significant differences (*P*<0.05).

To directly investigate *CitAco3* function, we introduced a cDNA, under the control of the constitutive CaMV 35S promoter, into citrus leaves and fruits using *Agrobacterium*-mediated transient transformation (Hellens *et al.*, 2005). Compared with the control (empty vector), transient overexpression of *CitAco3* significantly reduced the citric acid content in citrus leaves and fruits. In leaves transformed with *CitAco3* or the empty vector, citric acid contents were 1.16 and 1.74 mg g^−1^, respectively (Fig. 2A). Similar results were observed in citrus fruits, where transient overexpression of *CitAco3* significantly reduced citric acid content to 12.11 mg g^−1^, compared with the empty vector, at 15.52 mg g^−1^ (Fig. 2B).

### In vivo *regulatory effects of transcription factors the on* CitAco3 *promoter*

**Fig. 3.**
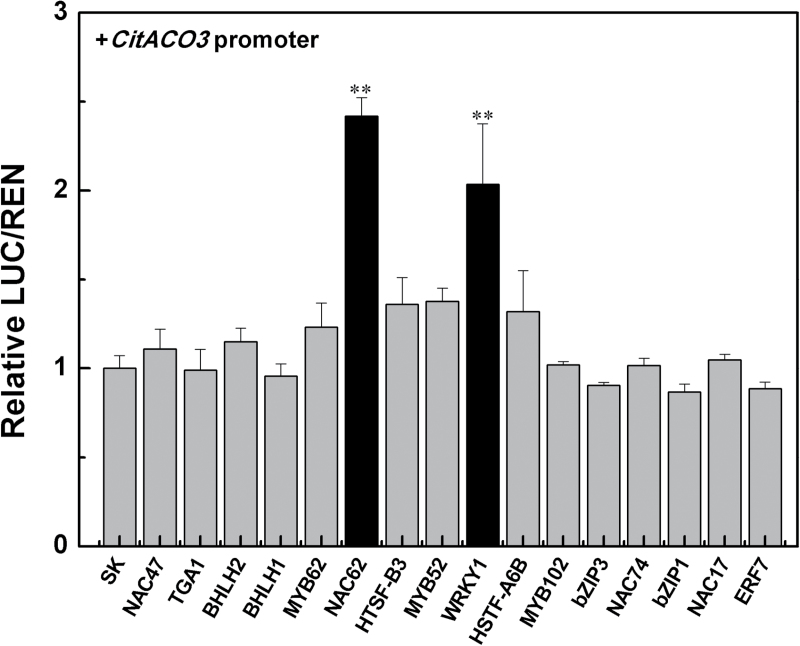
*In vivo* interaction of transcription factors with the promoter of the *CitAco3* gene from Ponkan fruit. *In vivo* associations of the transcription factors and promoter were obtained from transient expression assays in tobacco leaves. The ratio of firefly luciferase (LUC) and renilla luciferase (REN) of the empty vector (SK) plus promoter was set at 1. Error bars indicate SE from at least five replicates. **Significant differences (*P*<0.01).

In order to study the transcriptional regulation of *CitAco3*, we searched the RNA-Seq data from our previous report (Lin *et al.*, 2015) to identify 16 transcription factors whose abundance was highly correlated with *CitAco3* (Table 1). Dual luciferase assays indicated that in the presence of *CitNAC62* or *CitWRKY1*, *CitAco3* promoter activity was significantly enhanced, with approximately 2.4- and 2.0-fold induction, respectively (Fig. 3).

**Fig. 4.**
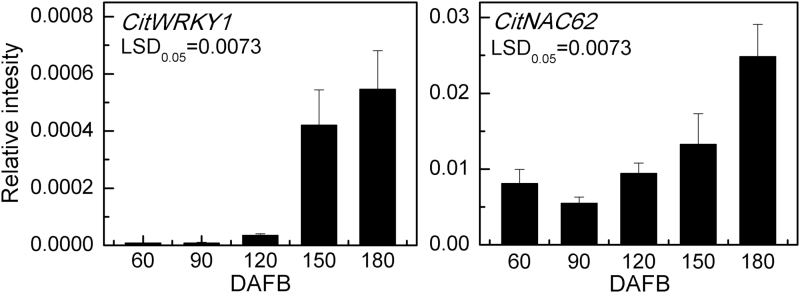
Expression of the *CitWRKY1* and *CitNAC62* genes in flesh of Ponkan fruits during fruit development, DAFB, days after full blossom. Error bars represent SE (*n*=3).

Analysis of *CitNAC62* and *CitWRKY1* expression indicated that both transcription factors had expression patterns similar to that of *CitAco3*, being more abundant at the late stages of fruit development (Fig. 4).

### *Subcellular localization and interaction of* CitNAC62 *and* CitWRKY1

**Fig. 5.**
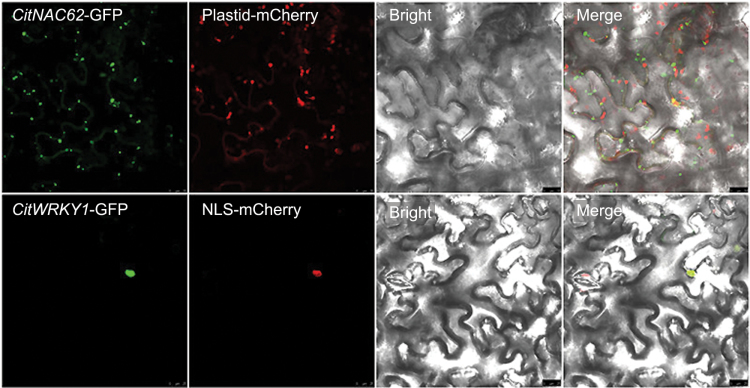
Subcellular localization of *CitNAC62*-GFP and *CitWRKY1*-GFP in tobacco leaves transformed by agroinfiltration. GFP fluorescence of *CitNAC62*-GFP and *CitWRKY1*-GFP is indicated. Bars=25 µm.

**Fig. 6.**
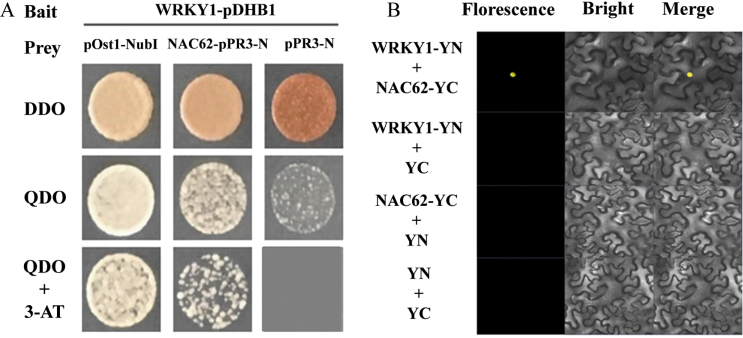
(A) Interaction between CitWRKY1 and CitNAC62 in yeast two-hybrid assays. Liquid cultures of double transformants were plated at OD_600_=0.1 dilutions on synthetic dropout selective media: (i) SD medium lacking Trp and Leu (DDO); (ii) SD medium lacking Trp, Leu, His and Ade (QDO); and (iii) SD medium lacking Trp, Leu, His, and Ade, and supplemented with 60 mM 3-amino-1,2,4-triazole (QDO+3AT). Protein–protein interactions were determined on QDO and QDO+3AT. pOst1-NubI, positive control; pPR3-N, negative control. (B) *In vivo* interaction between CitNAC62 and CitWRKY1, determined using BiFC. N- and C-terminal fragments of YFP (indicated on the figure as YN and YC) were fused to the C-terminus of CitNAC62 and CitWRKY1, respectively. Combinations of YC or YN with the corresponding CitNAC62 and CitWRKY1 constructs were used as negative controls. Fluorescence of YFP represents protein–protein interaction. Bars=50 µm.

To visualize the subcellular locations of the two transcription factors, we performed a subcellular localization assay in tobacco leaves by using GFP tagging. *CitWRKY1* gave strong signals in the nucleus (Fig. 5); *CitNAC62* was not located in the nucleus and the signals indicated that its subcellular location was within plastids (Fig. 5). Despite the different locations of the two transcription factors, protein–protein interactions were observed between CitNAC62 and CitWRKY1 in yeast two-hybrid assays (Fig. 6A). This interaction was also verified by bimolecular fluorescence complementation assays (BiFC) using tobacco leaves. The results showed that negative combinations, such as YFP^N^/CitNAC62-YFP^C^, CitWRKY1-YFP^N^/YFP^C^_,_ and YFP^N^/YFP^C^ did not produce any detectable fluorescence signal, while co-expression of CitNAC62-YFP^C^ and CitWRKY1-YFP^N^ gave strong signals in the nucleus (Fig. 6B).

### *Citric acid content is negatively regulated by* CitNAC62 *and* CitWRKY1

**Fig. 7.**
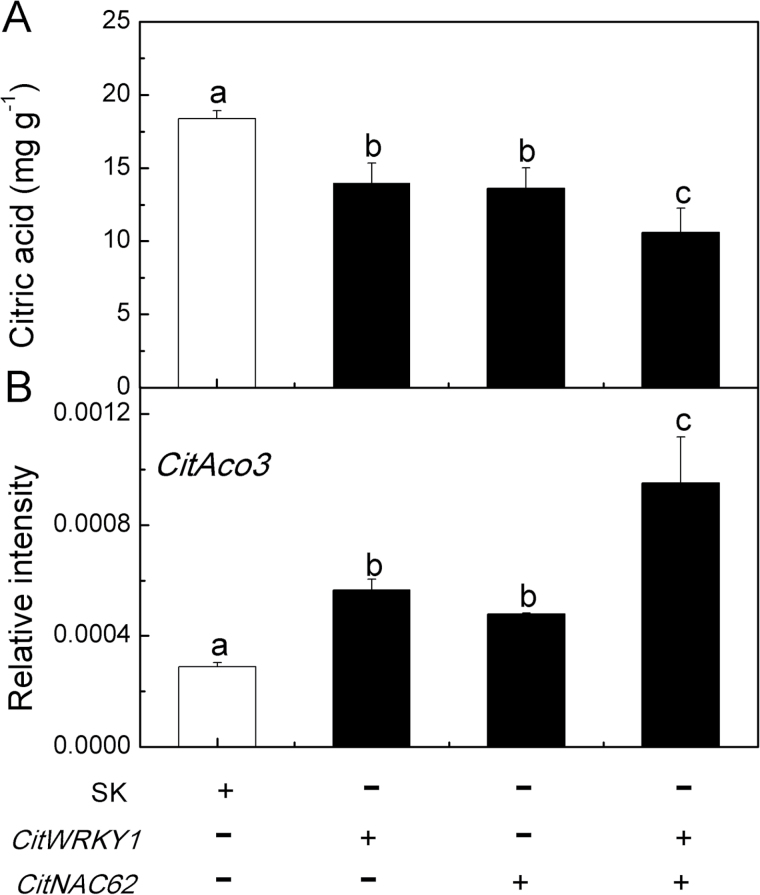
Effect of transient overexpression of *CitNAC62* and *CitWRKY1* on (A) citric acid content and (B) expression of *CitAco3* in citrus fruits. *CitNAC62* and *CitWRKY1* genes were driven by the CaMV 35S promoter. SK represents empty vector. Citric acid was analyzed at 5 d after infiltration. Error bars represent SE (*n*=3).

*CitNAC62* and *CitWRKY1*, under the control of the CaMV 35S promoter, were introduced into citrus fruits using *Agrobacterium*-mediated transient transformation (Hellens *et al.*, 2005). Compared with an empty vector control, transient overexpression of *CitNAC62* and *CitWRKY1* significantly decreased the citric acid content in citrus fruits, with values of 13.61 and 13.98 mg g^−1^, respectively, compared with 18.37 mg g^−1^ for the empty vector control. Transient overexpression of the combination of *CitNAC62* and *CitWRKY1* resulted in lower citric acid content in citrus fruits, at 10.59 mg g^−1^ (Fig. 7A). Transient overexpression of *CitNAC62* or CitWRKY1 significantly increased *CitAco3* abundance (Fig. 7B). Furthermore, co-introduction of both *CitNAC62* and *CitWRKY1* resulted in even lower citric acid content and higher *CitAco3* expression (Fig. 7), indicating that the two transcription factors can act in combination to increase the level of *CitAco3* and decrease the citric acid content.

## Discussion

### CitAco3 *is a contributor to citric acid degradation*

Multiple reports have correlated gene expression with citric acid degradation in citrus fruit, including an aconitase gene, *CitAco3* (Chen *et al.*, 2013; Lin *et al.*, 2015). In the present study, the association of *CitAco3* and citric acid degradation was confirmed during Ponkan fruit development. However, owing to the difficulty of transformation in perennial fruit such as citrus, validation of the function of *CitAco3* has not been performed. With the development of a citrus trans-transformation system (Shen *et al.*, 2016; Yin *et al.*, 2016), we have now shown that transient overexpression of *CitAco3* led to lower citric acid content in citrus fruit and leaves, supporting a role for *CitAco3* in citric acid degradation. A similar function for *Aco3* has been reported in other plants, including Arabidopsis (Hooks *et al.*, 2014) and tomato (Morgan *et al.*, 2013). The present results support the potential role of *CitAco3* in citric acid degradation in citrus fruit.

### *Transcription factors* CitNAC62 *and* CitWRKY1 *up-regulate* CitAco3 *transcript abundance and decrease citric acid content*

In recent years, molecular and genetic studies have identified numerous transcription factors participating in the regulation of fruit quality (Xie *et al.*, 2016). For instance, *AP2/ERF* transcription factors are involved in citrus fruit degreening (*CitERF13*; Yin *et al.*, 2016) and volatile metabolism (*CitAP2.10*; Shen *et al.*, 2016); and *PavMYB10.1* is involved in anthocyanin biosynthesis in sweet cherry fruit (Jin *et al.*, 2016). For organic acid metabolism, an EIN3-like transcription factor was characterized as the regulator of the ALMT1-like protein in apples (Bai *et al.*, 2015). In addition, MdMYB1 in apple fruits could activate the expression of two vacuolar H^+^-ATPase genes (*MdVHA-B1* and *MdVHA-B2*), affecting malate accumulation (Hu *et al.*, 2016). However, transcriptional regulation of citrate-related genes is largely unexplored. Here, we showed that *CitNAC62* and *CitWRKY1* regulate *CitAco3* transcript abundance *in vivo*. Furthermore, transient overexpression of *CitNAC62* and *CitWRKY1* resulted in lower citric acid content in citrus fruit. Thus, we propose that *CitNAC62* and *CitWRKY1* are negative regulators of citric acid content, acting via up-regulation of the *CitAco3* promoter.

### Protein–protein interaction between CitNAC62 and CitWRKY1 also involves translocation

An interesting finding was the protein–protein interaction between CitNAC62 and CitWRKY1, which suggests that the complex of transcription factors may contribute to citric acid degradation. Protein–protein interaction between transcription factors has been widely demonstrated in many plants, including fruit species. For example, MYBs, bHLHs, and WD40s have been shown to act together in a ternary regulatory MYB-BHLH-WD40 complex in order to regulate target genes, especially in anthocyanin biosynthesis (Schaart *et al.*, 2013), and EjAP2-1 regulates lignin biosynthesis via interaction with EjMYB1 and EjMYB2 in loquat fruits (Zeng *et al.*, 2015). However, such an interaction has not been reported for the regulation of organic acid metabolism. Thus, the impact of the interaction of CitNAC62 and CitWRKY1 on citric acid degradation may be only moderate (according to the transient overexpression data), but the interaction provides a novel clue about citric acid regulation.

BiFC analysis indicated that interaction between CitNAC62 and CitWRKY1 happens in the nucleus, but subcellular localization analysis indicated that only CitWRKY1, and not CitNAC62, is located within the nucleus. These results suggested that CitWRKY1 may translocate CitNAC62 to the nucleus. Translocation of genes by protein–protein interactions plays important roles in plants. In Arabidopsis, AtEBP may move from the nucleus to the cytoplasm via protein–protein interaction with ACBP4 (Li *et al.*, 2008); in rice, OsSPX4 could prevent OsPHR2 from being targeted to the nucleus through its interaction with OsPHR2 when phosphate is sufficient (Lv *et al.*, 2014). The present findings suggest that translocation of CitNAC62 may also contribute to citric acid degradation; however, the specific role of this translocation requires further investigation. In particular, the role and mechanism of CitWRKY1 for translocation, as well as the triggers of translocation, are unclear, and it is important to evaluate the function of such translocation in citric acid degradation.

## Supplementary data

Supplementary data are available at *JXB* online.

Table S1. Primers for real-time quantitative PCR analysis.

Table S2. Primers used for amplification of the promoter of *CitAco3* and SK construction.

Table S3. Primers used in subcellular localization analysis.

Table S4. Primers for yeast two-hybrid and BiFC assays.

Table S5. Primers used in transient overexpression analysis.

## Supplementary Material

Supplementary Tables S1-S5

## Abbreviations:

BiFC: bimolecular fluorescence complementation
DAFB: days after full blossom
GABA: gamma-aminobutyric acid
LSD: least significant difference.
