# Supplementary material for: Citrus CitNAC62 cooperates with CitWRKY1 to participate in citric acid degradation via up-regulation of CitAco3
Source: J Exp Bot. 2017 Jun 15;68(13):3419–26. doi: 10.1093/jxb/erx187 (PMC5853897; doi:10.1093/jxb/erx187)
Supplement: Supplementary Tables S1-S5 [file erx187_suppl_supplementary_tables_s1_s5.pdf]

Citrus CitNAC62 cooperates with CitWRKY1 to participate in citric acid degradation via up-regulation on *CitAco3*

Shao-jia Li[1,2,3,#], Xue-ren Yin[1,2,3,#] , Wen-li Wang[1,2] , Xiao-fen Liu[1,2] , Bo Zhang[1,2,3], Kun-song Chen[1,2,3,*]

Table S1 Primers for real-time quantitative PCR analysis

Gene	Forward primer (5’-3’)	Reverse primer (5’-3’)
CitAco3	GCATGAGGCATGAGGATTC	TTGGCCAAAAGAAAAATGAA
CitNAC62	CCTTTGTTTGTATAATTTTCCCATC	GGATCAAAATACAAAGTTAGGTTCA
CitWRKY1	GGCCAAGAAAATGAACTTGTCA	AGAGACCACCGTTCAGGGTAAC
F-CitNAC62	AATCTTGCGAGCAGGACGTT	TGTGGATTGCGCATCCTAGT
F-CitWRKY1	GCTCAACCGAAAGGAGGGAA	TTGAGCTGCTGCACCGATAA
CitActin	CATCCCTCAGCACCTTCC	CCAACCTTAGCACTTCTCC

F-CitNAC62 and *F-CitWRKY1* were used for gene expression in transient over-expressed fruit pulp.

Table S2 Primers used for amplification of the promoter of *CitAco3* and SK construction

Gene	Forward primer (5’-3’)	Reverse primer (5’-3’)
CitAco3-Luc	CAGGCGGCCGCCAACCACCAACATGATAACTGTGAC	AATATCCATGGCGACGTCGTTTCTGGAAATTTAG
CitBHLH1-SK	TTGGCGGCCGCATGGGTTCTGAGTCTTCTGCTTC	GGTACTAGTTTATTTGTTTCTATCAGCATTTTCC
CitNAC62-SK	GTTGCGGCCGCATGGCAGTGTTGTCATTGAATTCACT	AAGACTAGTTCAAAAATTAAGGCAAAGACATCTCCATG
CitMYB52-SK	GTGGCGGCCGCATGGAGGATTCAGGAGCTGGTTCTA	TTTACTAGTTCAAGAAGTGATACCGACACCGAGGA
CitHSTFA6B-SK	GTGGCGGCCGCATGCATCCAACAGGTAGAGTCGA	TCCACTAGTTTACTTTGGACTCGAACTCAGGAAAC
CitbZIP1-SK	GAGGCGGCCGCATGGTGGCACCAGAGCCGGAAACGC	TCCACTAGTTTACAATGAGCAACTTAAATTTCTTC
CitMYB102-SK	GAGGCGGCCGCATGGGAAGGCCACCAAGCTCCGA	TGTACTAGTTTATACAAGTCCAATCGCATCCAA
CitWRKY1-SK	GAGGCGGCCGCATGGACTGGGATTTGCAAGCCAT	TCCACTAGTTCAGAGACCACCGTTCAGGGTAACA
CitNAC74-SK	TAAGCGGCCGCATGGGGCTAAGAGATATTGGAGCT	TCCACTAGTTCATAGGAAAACCATGCTGTTATCC
CitNAC17-SK	GTGGCGGCCGCATGAAGGTGACTTCAGAAAACGTGTGG	TACACTAGTTCAAGAGGAGATGCAACTGCCCAAGACTC
CitHSTFB3-SK	ATTGCGGCCGCATGGAGGCTACAAATAATATTA	AGACTGCAGTTATTTGCATGATTGAGATAGTAA
CitMYB62-SK	GGGGCGGCCGCATGCACACAATGAGAGCAGCAAG	TGCACTAGTTTACTCCCTAAGCTGCCATATGTC
CitbZIP3-SK	AAAGCGGCCGCATGTCCGATCCGCTCCTCGCCGAAGCA	TTTACTAGTTTAACTGGTCACTAGATGAAGACCAGATC
CitERF7-SK	CTCGCGGCCGCATGAGGAGGGCGAGAGGAGCC	CAACTGCAGTCAGAGACATAAAGCGGTGCACT
CitBHLH2-SK	GTGGCGGCCGCATGGATTCAAGTACTAATCATAATT	GGACTGCAGTCAAACAATTTGATTCTCGACTGGTTT

CitNAC47-SK	TATGCGGCCGCATGGTTTGCATAAAGAACCCGGAA	TTCCTGCAGTCATCCTTGAAACTGAAGATGTGGGC
CitTGA1-SK	GTTGCGGCCGCATGAACTCTCCGTCCACCCAGTAT	GCTACTAGTCTAGGTGGGCTCCCGAGGACGATTAG

Table S3 Primers used in subcellular localization analysis

Gene	Forward primer (5’-3’)	Reverse primer (5’-3’)
CitNAC62-GFP	TTCGGTACCATGGCAGTGTTGTCATTGAATTCACT	CAAGTCGACTCAAAAATTAAGGCAAAGACATCTCCATG
CitWRKY1-GFP	TTTGGTACCATGGACTGGGATTTGCAAGCCAT	CATGTCGACACTGAGACCACCGTTCAGGGTAACA

Table S4 Primers for yeast two-hybrid and BiFC assays

Gene	Forward primer (5’-3’)	Reverse primer (5’-3’)
CitNAC62	GCAGAGTGGCCATTACGGCCATG	CTCGAGAGGCCGAGGCGGCCGAA
pPR3-N	GCAGTGTTGTCATTGAATTCACT	AATTAAGGCAAAGACATCTCCATG
CitWRKY1	AGAACGCGGCCATTACGGCCATG	CCGACATGGCCGAGGCGGC
PDHB1	GACTGGGATTTGCAAGCCAT	CCGACATGGCCGAGGCGGCCAA
CitNAC62	CCCAAATTCGCGACCGGTATGG	GAGACCACCGTTCAGGGTAACA
YC	CAGTGTTGTCATTGAATTCACT	TAAGGCAAAGACATCTCCATG
CitWRKY1	CCCAAATTCGCGACCGGTATG	GCTGCACGCTGCCACCGGTGA
YN	GACTGGGATTTGCAAGCCAT	GACCACCGTTCAGGGTAACA

Table S5 Primers used in transient overexpression analysis

Gene	Forward primer (5’-3’)	Reverse primer (5’-3’)
CitAco3-SK	ATGTATATTCCGAGTTCTTCAGCGTCTCGCGCACGC	TTACTGTTTAATCAGATTCCGGATAACATATGGAAG
CitNAC62-SK	GTTGCGGCCGCATGGCAGTGTTGTCATTGAATTCACT	AAGACTAGTTCAAAAATTAAGGCAAAGACATCTCCATG
CitWRKY1-SK	GAGGCGGCCGCATGGACTGGGATTTGCAAGCCAT	TCCACTAGTTCAGAGACCACCGTTCAGGGTAACA
